# The role of trauma experiences, personality traits, and genotype in maintaining posttraumatic stress disorder symptoms among child survivors of the Wenchuan earthquake

**DOI:** 10.1186/s12888-020-02844-1

**Published:** 2020-09-07

**Authors:** Yuwei Li, Qiuyue Lv, Bin Li, Dan Luo, Xueli Sun, Jiajun Xu

**Affiliations:** Mental Health Center, West China Hospital, Sichuan University, Chengdu, China

**Keywords:** Posttraumatic stress disorder, Children, Earthquake, Personality traits, Long-term potentiation

## Abstract

**Background:**

Posttraumatic stress disorder (PTSD) is the most prevalent type of psychiatric disorder among children after an earthquake. This study investigated the role of trauma experiences, personality traits, and genotype in the maintenance of PTSD symptoms.

**Methods:**

In a previous large-scale epidemiological investigation 1 year after the Wenchuan earthquake, 215 children with PTSD symptoms were selected at random with their blood samples collected. All of them were followed up, and their PTSD symptoms were assessed 3 years later. The adolescent version of the UCLA PTSD Reaction Index, the earthquake exposure scale, and the Junior Eysenck Personality Questionnaire were used to determine PTSD symptoms, trauma experiences, and personality traits, respectively. We sequenced candidate genes involved in the regulation of long-term potentiation via NMDA-type receptors to identify the related SNP variations.

**Results:**

Being trapped for a longer period of time, feeling one’s own or a family member’s life to be in danger, losing a close family member or friend, extraversion, neuroticism, TrkB, G72 and CNTF were found to be associated with the maintenance of PTSD symptoms.

**Conclusions:**

Experiences, personality traits, and genotype influenced the maintenance of PTSD in child survivors who were considered to be followed up without medicine. This result could help to identify potential targets for treatment and promote the rational allocation of medical resources.

## Background

Earthquakes are one of the most unpredictable natural disasters and have a massive influence on the psychological and physical state of survivors. Posttraumatic stress disorder (PTSD) is the most prevalent type of psychiatric disorder among earthquake survivors, and it can be defined as delayed but lasting psychological stress disorder [1]. It is also a disabling disorder related to high comorbidity rates of depression, anxiety, and suicidal ideation [2]. Recent studies have suggested that the point prevalence rate of PTSD among child survivors after earthquakes is 12.4–28.4% [3–5], and trauma could have a lasting impact on children’s developing brain and body [6].

Numerous empirical studies support the efficacy of trauma-focused psychological interventions [7, 8] and pharmacotherapy [9]. In clinical practice, people without treatment-seeking behavior [10] or enough medical care [11] have a limited chance of recovering from PTSD symptoms. Since the resources for intervention could be insufficient, it would be meaningful to investigate the maintenance factors for the PTSD patients who survived an earthquake and promote the rational allocation of medical resources.

Several studies have revealed that PTSD symptoms tend to heal spontaneously over time [12, 13]. This process can be influenced by several factors that can only be assessed based on data about the long-term outcomes of PTSD without treatment. Although extensive research listed age, gender, posttraumatic growth, and other traumas as significant influences on the recovery process of children [14–16], few writers have been able to draw on any systematic research into the impact of various factors on the maintenance of PTSD in children.

Previously published studies on the onset factors of PTSD have confirmed the influence of trauma experiences, personality traits, and genotype [4, 17, 18]. However, the maintenance factors of PTSD are rarely systematically evaluated. Along with the continuity of the occurrence and development process, the elements that affect the onset may relate to maintaining PTSD symptoms, and exploring the influencing factors in the emergence of PTSD is vital for maintaining factors. Trauma exposure experiences outside the realm of daily life evoke fear, helplessness, and horror, and PTSD is a psychiatric disorder that develops after individuals suffer from life-threatening traumatic events [19–22]. However, patients tend to manifest different symptoms of the disorder even after having the same experiences, which means that personality traits play an essential role in PTSD [23–25]. It is worth noting that the genotype is the same as personal traits among PTSD. In terms of molecular mechanisms, long-term potentiation (LTP) is dependent on NMDA receptors (NMDARs) in synapses, and it is the fundamental cellular correlate of learning and memory [26]. Synaptic plasticity, to which LTP pertains [27], plays a vital role in the developing brain and responds to a wide range of factors, such as life experiences [28]. All facts suggest that LTP is of concern in childhood. In addition, stress can impair LTP [29, 30], and the decrease in LTP induction in mice resulted in facilitated fear memories [31]. The findings of studies suggest that LTP takes part in the onset and maintenance of PTSD [32], and this process is adjusted by cytokines, such as BDNF and TrkB [26, 33–35]. The investigation of the genotype of related cytokines will significantly increase our knowledge of LTP in PTSD.

On May 12, 2008, a devastating earthquake (measuring 8.0 on the Richter scale) struck southwestern China and caused the extensive onset of psychiatric disorders among the survivors [36]. The symptoms emerged weeks or months after the traumatic events, and even 3 years later, the prevalence of PTSD remained at 10.3% [37]. Children were undoubtedly psychologically and physiologically affected by the earthquake. Due to the weak natural conditions and lack of local medical resources, it was difficult to provide effective treatment in time. The lack of treatment allowed the symptoms to run their natural course, and the follow-up of child survivors could fully assess the impact of various factors on the disease maintenance of PTSD. Accordingly, in this study, we aimed to investigate child survivors to explore the role of trauma experiences, personality traits, and genotype on the maintenance of PTSD symptoms. We hypothesized that trauma experiences, personality traits, and genotype are vital factors influencing the maintenance of PTSD symptoms, even when adjustments are made for other risk factors.

## Methods

### Participants and procedure

**Fig. 1:**
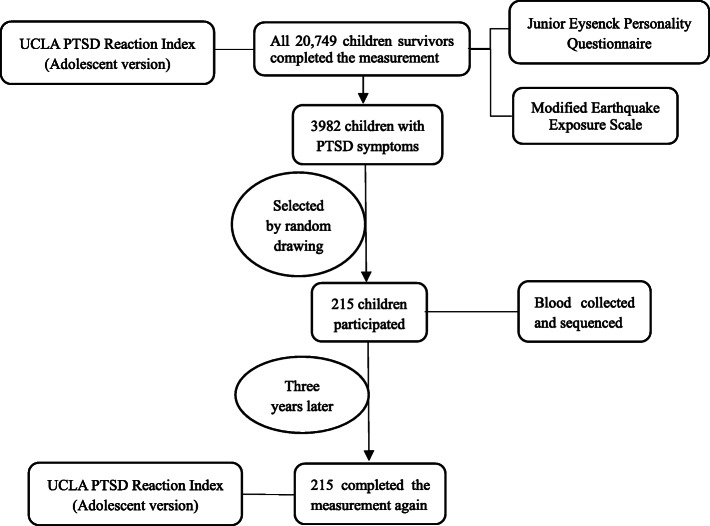
Sampling stages for child survivors in Qingchuan, Sichuan Province, following the Wenchuan earthquake

The children were recruited from schools in Qingchuan, Sichuan Province, from May to July 2009, approximately 1 year after the Wenchuan earthquake. The relatively stringent time horizon was executed because the manifestations in children would spontaneously change after the earthquake [38, 39]. A total of 20,749 children aged 7–15 years old in Qingchuan were thoroughly tested by the adolescent version of the UCLA PTSD Reaction Index, the earthquake exposure scale, and the Junior Eysenck Personality Questionnaire (JEPQ) by trained psychiatrists. According to the UCLA PTSD Reaction Index score, 3982 children had PTSD symptoms [4]. We randomly selected two villages out of the 31 townships in Qingchuan and recruited all the children with PTSD symptoms in the two towns as our subjects. A total of 215 children were selected, and there was no significant difference between the selection and nonselection groups. The blood of the selection group was collected at the beginning of their enrollment. After 3 years, all participants were then tested with the adolescent version of the UCLA PTSD Reaction Index again and assessed thoroughly for PTSD symptoms (Fig. 1).

All the surveys were conducted through face-to-face interviews by trained psychiatrists, psychologists, psychiatric nurses, and social workers. All participants and their parents or guardians provided informed consent and were informed of their rights to withdraw at any time. During the interviews, the child was assessed in school without being accompanied by parents or guardians. This procedure ensured that all questions were completed by themselves without interruptions.

### Measures

The adolescent version of the UCLA PTSD Reaction Index was used to evaluate symptoms of PTSD [40]. The index contains 20 items on a 5-point scale and is used to rate the frequency of symptom occurrence. It was translated into straightforward Chinese to assess children’s symptoms. The index could evaluate almost all the signs of PTSD, including re-experiencing, avoidance, increased arousal symptoms, numbness, and pessimism symptoms [41]. If the total score is equal to or higher than 36, the child is identified as having symptoms of PTSD [42].

The earthquake exposure scale is an earthquake-modified version of the PsySTART Rapid Triage System [22]. It includes ten yes-and-no questions through which the results are more natural to obtain. The questions were adapted from the prior earthquake exposure scales, which were drafted based on the Diagnostic and Statistical Manual of Mental Disorders A-1 and A-2 criteria for PTSD [43, 44]. The experiences could be separated into two categories. Half of the items are used to evaluate subjective factors, including having experienced extreme panic or fear and having felt others’ or a family member’s life to be in danger, while the others are used for objective factors, including the loss of a close family member or friend.

The Junior Eysenck Personality Questionnaire is a commonly used self-report questionnaire that was translated into Chinese and modified 30 years ago [45–48]. It is widely used in children aged 7 to 15 to assess their personality traits. The Chinese version of the JEPQ could evaluate four characteristics by 88 yes-or-no items. The four subscales are psychoticism (P), extraversion (E), neuroticism (N), and lie, with five levels in each. For example, the P subscale is divided into five levels: psychoticism (T < 38.5), moderate psychoticism (38.5 ≤ T < 43.3), intermediate (43.3 ≤ T ≤ 56.7), moderate socialization (56.7 < T ≤ 61.5) and socialization (T > 61.5). The other subscales are divided similarly.

### SNP selection and genotyping

There is some evidence that PTSD may be related to LTP [49]. According to the literature search, candidate genes include AKT1, APP, BDNF, TrkB, NTF3, BMP, G72, CNTF, S100B, NGF, EGF, and PSEN2, and all of them participate in regulating LTP via NMDAR. We searched known SNPs from databases (NCBI and HapMap) and genotyped ten DNA samples from each group. Genomic DNA was extracted from whole blood samples using a TIANamp Blood DNA Kit (DP318–03, TIANGEN, Beijing), and SNPs were genotyped with the SEQUENOM MassARRAYiPLEX platform. The assay consists of an initial locus-specific PCR, followed by single-base extension and matrix-assisted laser desorption/ionization-time of flight mass spectrometry to identify the SNP allele. Using Wang’s method [50] and analyzing twenty DNA samples, 28 SNPs were selected with allele frequencies higher than 5%. Finally, all blood samples were genotyped on 28 SNPs.

### Statistical analyses

Statistical analyses were performed by R, and packages of psych, MASS, AER, and leaps were used. The frequencies of all variables were calculated. The age and the scores of the adolescent version of the UCLA PTSD Reaction Index were described by the mean and standard deviation, and the others were described by counts and ratios. The chi-square test, Student’s t-test, and the Wilcoxon rank-sum test were used to analyze the difference between the two groups. Furthermore, logistic regression analysis was used to adjust the influence of demographic factors and the severity of symptoms, and multinomial logistic regression analysis was used to identify the role of trauma experiences, personality traits, and genes. All factors were chosen in multinomial logistics regression, including the demographic data (sex, age, residence, removal or not), the scores of three scales, and all the results of the SNP test. R automatically searched for the model with the highest R^2^, and the factors were selected by the software. The new multinomial logistic regression model had the factors chosen by the software, and this procedure yielded odds ratios with 95% confidence intervals for each variable. Finally, R calculated the relative importance of predictor variables. All tests were two-tailed, and those that had *p*-values of 0.05 or lower were treated as statistically significant in all data analyses.

## Results

### Demographic and clinical characteristics

The demographic information and clinical characteristics of all 215 participants in this study are summarized in Table 1. The children were divided into two groups. One group consisted of children who did not recover from PTSD 3 years after the earthquake, while the children in the other group did. Compared with the children who did not recover from PTSD symptoms, the recovered group tended to be younger (*p* < 0.05), have lower avoidance scores (p < 0.05) and have lower total UCLA-PTSD RI scores (*p* < 0.01).

**Table 1:** Demographic and clinical characteristics of the participants

	Nonrecovered(***N*** = 155)	Recovered(***N*** = 60)	p	Adjusted p
**Demographic factors**				
Boys	88 (56.8%)	30 (50%)	0.458	–
Age	10.58 ± 1.52	10.08 ± 1.52	**0.034**	**–**
Residence: Town	3 (1.9%)	3 (5.0%)	0.446	–
Move to another area after earthquake	60 (38.7%)	19 (31.7%)	0.422	–
**Earthquake exposure**				
Saw anyone dead or injured (A2)	120 (77.4%)	42 (70.0%)	0.339	0.711
Felt others’ panic (A3)	137 (88.4%)	55 (91.7%)	0.651	0.100
Trapped for a longer period of time (A4)	77 (49.7%)	8 (13.3%)	**<0.001**	**<0.001**
Felt one’s own or a family member’s life to be in danger (A5)	128 (82.6%)	56 (93.3%)	0.072	**0.039**
Felt unable to escape from the disaster (A6)	114 (73.5%)	34 (56.7%)	**0.026**	0.061
Felt extreme panic or fear (A7)	144 (92.9%)	53 (88.3%)	0.418	0.460
Lost a close family member or friend (A8)	55 (35.5%)	10 (16.7%)	**0.011**	**0.013**
Had a close family member or friend injured (A9)	85 (54.8%)	34 (56.7%)	0.929	0.624
Lost home or important belongings (A10)	126 (81.3%)	46 (76.7%)	0.569	0.520
Injured (A11)	27 (17.4%)	6 (10.0%)	0.253	0.216
**JEPQ**				
Psubscales			0.577	0.355
Psychoticism	25 (16.1%)	6 (10.0%)		
Moderate psychoticism	12 (7.7%)	8 (13.3%)		
Intermediate	73 (47.1%)	27 (45.0%)		
Moderate socialization	35 (22.6%)	13 (21.7%)		
Socialization	10 (6.5%)	6 (10.0%)		
Esubscales			**< 0.001**	**< 0.001**
Extraversion	13 (8.4%)	14 (23.3%)		
Moderate extraversion	18 (11.6%)	16 (26.7%)		
Intermediate	76 (49.0%)	22 (36.7%)		
Moderate introversion	21 (13.5%)	6 (10.0%)		
Introversion	27 (17.4%)	2 (3.3%)		
Nsubscales			**< 0.001**	**< 0.001**
Neuroticism	22 (14.2%)	2 (3.3%)		
Moderate neuroticism	31 (20.0%)	6 (10.0%)		
Intermediate	79 (51.0%)	23 (38.3%)		
Moderate stability	9 (5.8%)	11 (18.3%)		
Stability	14 (9.0%)	18 (30.0%)		
**UCLA-PTSD RI**				
Re-experiencing	12.16 ± 2.61	11.80 ± 2.40	0.336	–
Avoidance	17.12 ± 3.41	16.02 ± 2.83	**0.017**	**–**
Increased arousal	13.66 ± 3.06	13.32 ± 2.31	0.378	–
Total score	42.94 ± 5.63	41.13 ± 3.84	**0.008**	**–**

For earthquake experience, the ratio of having trapped for a longer period of time was 49.7 and 13.3% for the nonrecovered and the recovered groups, respectively. The rates of feeling unable to escape from the disaster and having lost a close family member or friend were significantly different between the two groups.

With respect to the JEPQ, two groups showed significant differences on the E subscale (*p* < 0.001) and the N subscale (p < 0.001), and the recovered group scored higher on the E subscale and lower on the N subscale.

Taking the demographic factors and the UCLA-PTSD RI as the covariance, there was a significant difference between the two groups on earthquake exposure and the JEPQ. The result of the logistic regression analysis showed that the recovered group had a greater feeling that one’s own or a family member’s life was in danger (*p* < 0.05), but the other results were similar to those of prior studies.

### Distribution of genotypes

In the case of SNPs related to LTP, the results are presented in Table 2. We found that they were basically the same in the two groups, and the *p*-values were 0.067–0.916. After adjustments were made for the influence of demographic factors and the severity of symptoms, the results showed that TrkB (rs920776) was significantly different between the two groups (*p* < 0.05).

**Table 2:** The distribution of genotypes of all SNPs: a) 0 represents the common homozygotes; b) 1 represents the heterozygotes; and c) 2 represents rare homozygotes

Gene	SNP ID symbol	Nonrecovered(N = 155)			Recovered(N = 60)			χ^2^	P*	p**
		0^a^	1^b^	2^c^	0	1	2			
PS2	rs1046240	60 (38.7)	78 (50.3)	17 (11.0)	26 (43.3)	26 (43.3)	8 (13.3)	0.876	0.645	0.555
	rs1295645	9 (5.8)	57 (36.8)	89 (57.4)	2 (3.3)	18 (30.0)	40 (66.7)	1.703	0.427	0.182
	rs8383	16 (10.3)	83 (53.5)	56 (36.1)	8 (13.3)	26 (43.3)	26 (43.3)	1.830	0.401	0.456
NOS1	rs1047735	42 (27.1)	77 (49.7)	36 (23.2)	14 (23.3)	39 (65.0)	7 (11.7)	5.007	0.082	0.382
TrkB	rs1047896	127 (81.9)	28 (18.1)	0 (0.0)	44 (73.3)	15 (25.0)	1 (1.7)	4.026	0.134	0.140
	rs1624327	3 (1.9)	46 (29.7)	106 (68.4)	2 (3.3)	19 (31.7)	39 (65.0)	0.494	0.781	0.748
	rs2013566	5 (3.2)	53 (34.2)	97 (62.6)	2 (3.3)	14 (23.3)	44 (73.3)	2.401	0.301	0.171
	rs7020204	4 (2.6)	53 (34.2)	98 (63.2)	2 (3.3)	15 (25.0)	43 (71.7)	1.714	0.425	0.323
	rs920776	5 (3.2)	57 (36.8)	93 (60.0)	1 (1.7)	13 (21.7)	46 (76.7)	5.268	0.072	**0.025**
AKT1	rs1130233	27 (17.4)	70 (45.2)	58 (37.4)	7 (11.7)	33 (55.0)	20 (33.3)	1.978	0.372	0.729
G72	rs2391191	45 (29.0)	84 (54.2)	26 (16.8)	18 (30.0)	30 (50.0)	12 (20.0)	0.412	0.814	0.647
	rs1421292	53 (34.2)	80 (51.6)	22 (14.2)	23 (38.3)	30 (50.0)	7 (11.7)	0.426	0.804	0.615
	rs3916965	26 (16.8)	83 (53.5)	46 (29.7)	12 (20.0)	30 (50.0)	18 (30.0)	0.360	0.835	0.617
	rs3916966	46 (29.7)	83 (53.5)	26 (16.8)	18 (30.0)	30 (50.0)	12 (20.0)	0.360	0.835	0.596
	rs3918341	42 (27.1)	85 (54.8)	28 (18.1)	20 (33.3)	28 (46.7)	12 (20.0)	1.220	0.543	0.872
	rs3918342	32 (20.6)	87 (56.1)	36 (23.2)	11 (18.3)	34 (56.7)	15 (25.0)	0.175	0.916	0.880
	rs778294	107 (69.0)	41 (26.5)	7 (4.5)	42 (70.0)	18 (30.0)	0 (0.0)	2.914	0.233	0.464
	rs947267	28 (18.2)	77 (50.0)	49 (31.8)	8 (13.3)	33 (55.0)	19 (31.7)	0.814	0.666	0.645
S100B	rs2300403	43 (27.7)	82 (52.9)	30 (19.4)	18 (30.0)	28 (46.7)	14 (23.3)	0.741	0.690	0.856
CNTF	rs2515362	12 (7.7)	71 (45.8)	72 (46.5)	11 (18.3)	25 (41.7)	24 (40.0)	5.105	0.078	0.070
APP	rs2830102	5 (3.2)	56 (36.1)	94 (60.6)	4 (6.7)	19 (31.7)	37 (61.7)	1.478	0.478	0.639
DISC1	rs4658971	7 (4.5)	56 (36.1)	92 (59.4)	2 (3.3)	24 (40.0)	34 (56.7)	0.372	0.830	0.731
	rs821597	30 (19.4)	82 (52.9)	43 (27.7)	13 (21.7)	28 (46.7)	19 (1.7)	0.675	0.713	0.976
	rs821616	122 (78.7)	28 (18.1)	5 (3.2)	50 (83.3)	8 (13.3)	2 (3.3)	0.695	0.706	0.510
BDNF	rs6265	46 (29.7)	73 (47.1)	36 (23.2)	15 (25.0)	31 (51.7)	14 (23.3)	0.521	0.771	0.630
NGF	rs6330	5 (3.2)	47 (30.3)	103 (66.6)	1 (1.7)	26 (43.3)	33 (55.0)	3.430	0.180	0.066
NTF3	rs6332	37 (23.9)	83 (53.5)	35 (22.6)	18 (30.0)	30 (50.0)	12 (20.0)	0.871	0.647	0.489
PS1	rs7523	106 (68.4)	40 (25.8)	9 (5.8)	38 (63.3)	22 (36.7)	0 (0.0)	5.418	0.067	0.635

### Maintaining factors of PTSD symptoms

**Fig. 2:**
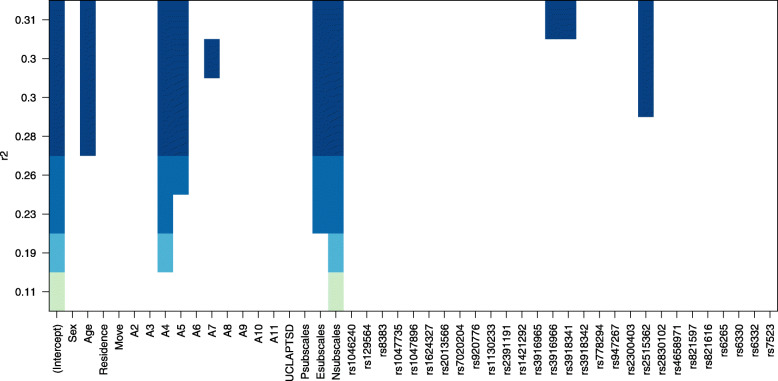
Plot for all subset regressions

**Fig. 3:**
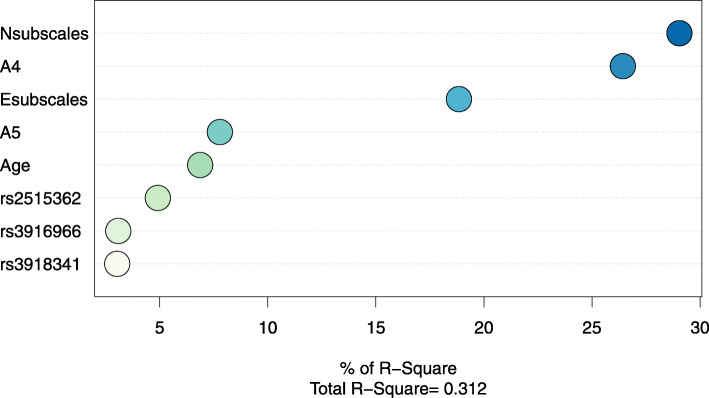
Relative importance of predictor variables

The best regression model with the highest R^2^ was made with eight variables, including being trapped for a longer period of time, feeling one’s own or a family member’s life to be in danger, Esubscales, Nsubscales, and SNPs (rs3916966, rs3918341, rs2515362) (Fig. 2 and Table 3). Protective factors are A5, Esubscales, and rs3916966, indicating that the child felt his own or a family member’s life to be in danger, typically characterized by extraversion or with AA on rs3916966 seeming to recover from PTSD symptoms more quickly. Age, A4, Nsubscales, rs3918341, and rs2515362 were found to be risk factors indicating being an older child, being trapped for a longer period of time, being typically characterized by neuroticism, and having AA on rs3918341 and rs2515362 suffering more prolonged symptoms. Among multiple factors, the relative importance of predictor variables was calculated. The results showed that personality traits and traumatic experiences have the most significant influence on the maintenance of PTSD symptoms among child survivors of the Wenchuan earthquake (Fig. 3).

**Table 3:** Logistic regression analysis of the factors selected by all-subset regression

	OR	p	95%CI
Age	0.756	0.021	0.593,0.957
A4	0.166	< 0.001	0.061,0.398
A5	6.023	0.006	1.836,24.776
Esubscales	1.865	0.001	1.304,2.760
Nsubscales	0.469	< 0.001	0.319,0.667
Rs3916966	12.706	0.048	1.458,310.089
Rs3918341	0.088	0.055	0.004,0.734
Rs2515362	0.509	0.020	0.283,0.890

## Discussion

This study was conducted among participants who underwent a natural, destructive disaster in a developing country. Due to the harsh natural environment, poor traffic conditions, and unbalanced medical conditions, these affected children went through a series of naturally developing processes of recovery from the post-earthquake stress reaction. That study explored the natural restoration processes of PTSD without treatment and found that demographic data, trauma experiences, personality traits, and genotype have a vital influence on the maintenance of PTSD symptoms.

There is some evidence to suggest the effect of age on PTSD symptoms. For example, a meta-analysis of 53 studies conducted by Tang found that older age was a significant predictor of children’s PTSD [51]. We held the same views. This finding may be explained by changes in resilience, but further studies are needed to confirm this hypothesis [52]. Regarding gender, a correlation was found between female gender and a higher risk of PTSD [37, 53–57]. In contrast, there was no significant relationship between gender and the maintenance of symptoms. According to research purposes, the effect of demographic factors and the severity of symptoms were eliminated in the following analysis.

The experience of trauma, which has a significant impact on the initiation and development of PTSD, could be divided into two groups: A) objective experiences, including actual reality and losing a family member, and B) subjective experiences, including personal feelings, intense fear, helplessness, or horror [58, 59]. There was some evidence to suggest that both subjective and objective earthquake exposure experiences could influence the development of PTSD symptoms [20, 60, 61]. On the other hand, we further confirmed their effects on maintenance symptoms and found that one subjective experience, namely, feeling one’s own or a family member’s life to be in danger, was a protective factor, and two objective experiences, namely, being trapped for a longer period of time and having lost a close family member or friend, were risk factors for the maintenance of PTSD symptoms. This inconsistency may be due to the differences in duration. Subjective exposure did not always represent objective reality, and measures such as spending time with others would help children feel safe with the dissipation of their undesirable feelings. By contrast, PTSD symptoms caused by objective exposure could last longer and require more attention.

Personality traits are conceptualized as dimensions of individual differences in tendencies to show consistent patterns of thoughts, feelings, and contexts [62]. Numerous models are used to describe personality traits, the most common of which are neuroticism, extraversion, and psychoticism. There were several differences in personality traits between the recovered and nonrecovered groups, such as neuroticism and extraversion. The findings of this study showed that neuroticism tends to be a risk factor with all elements under consideration. In the same way, the evidence from previous studies suggested that an increased level of neuroticism indicates a higher risk of developing PTSD and could be used as a critical predictor [23, 63, 64]. This phenomenon may result from the character of neuroticism. For example, one with a stronger trait of neuroticism often seems moody and excitable, and after a series of mishaps, children with unstable emotions usually develop psychiatric symptoms more easily and have a harder time recovering. Extraversion can be defined as positive emotional aspects interpersonally and socially and is generally seen as a factor strongly related to numerous psychological illnesses [65]. In their useful study, Nenad found that PTSD symptoms were negatively associated with extraversion [66]. Likewise, extraversion helped to alleviate PTSD symptoms in our results. There are several possible explanations for this result. Children with a higher trait of extraversion tend to be high in gregariousness, activity, and enthusiasm [67]. They are usually more able to relieve their emotions and find social support, which means that they can alleviate PTSD symptoms as soon as possible.

Another significant aspect of the effect factor is hereditary. This study focused on LTP, which is induced in a variety of brain regions with diverse simulation protocols and requires activation by NMDARs. NMDARs are vital mediators of excitatory synaptic transmission and are adjusted by numerous factors, such as TrkB, G72, and CNTF [68, 69]. The evidence from this study showed that TrkB (rs920776) was differentially distributed between the recovered group and the nonrecovered group within 3 years. TrkB is a single transmembrane receptor of BDNF and is essential for the formation of memories [70]. The BDNF pathway was assumed to play a role in several psychiatric disorders, including PTSD [71]. There is some evidence to suggest that enhanced levels of TrkB during fear memory consolidation are associated with long-lasting fear memory [72]. Moreover, recent research has revealed that BDNF-TrkB is a protective factor against PTSD. For example, a decrease in its signaling is likely a contributing factor in PTSD [73, 74], and BDNF-TrkB signaling might be a novel therapeutic strategy for impaired fear memory extinction by stress [75]. Previous studies have suggested a relationship between TrkB and PTSD, and we further strengthened this relationship. We found that TrkB is a contributory factor to the maintenance of PTSD, which could be caused by its participation in support of impaired contextual fear learning and fear extinction [73]. In addition, after other factors were excluded, G72 (rs3916966, rs3918341) and CNTF (rs2515362) tended to be influencing factors. The G72 genes are located on chromosome 13q33 and encode a primate-specific protein named pLG72, which is known as an interacting partner of flavoenzyme D-amino acid oxidase that could degrade the key endogenous co-agonist of NMDAR [76–78]. Previous researchers found evidence of the association between G72 polymorphisms, including rs3918342 and rs3696165, and schizophrenia, bipolar disorder, and major depressive disorder [79–83]. However, the relationship between G72 and PTSD has yet to be reported. CNTF can influence several types of peripheral and central neurons [84, 85], reduce synaptic depression during repetitive stimulation [86] and protect neurons from hypoxic injury [87]. CNTF levels increased in major depressive disorder, and CNTF was seen as a factor related to mental disease [88]. In addition, CNTF influenced stress-induced cortical norepinephrine synthesis, ensuing neuronal excitation and behavioral stereotypes [89], which may build a bridge between trauma experience and PTSD.

It is worth noting that among all probable factors, including demographic factors, the severity of symptoms, personality traits, trauma experiences and genes, personality traits, exposure experiences, and age played the most crucial role in the maintenance of PTSD. On the other hand, the currently known related genes showed limited influence on the maintenance of PTSD. This result may be caused by the small impact of single SNPs or the mediation of gene expression. A single SNP will not be enough to influence PTSD, but it probably sets up the genomic precondition, while personality traits, experiences, and the subsequent environment lead in the same direction. The interaction between the environment and genotype should also be considered, and other genes should be included to build the formula of the prediction model. This approach will more clearly demonstrate the influence of genes.

This study had some limitations. First, since the participants were selected among survivors, the sample sizes of each group were unbalanced. Expanding the sample quantity may solve this problem. Second, this study followed up for 3 years, during which some of the participants recuperated. Follow-up of all patients until recovery would be more productive and informative. Third, this study measured the children’s personality traits after the earthquake, which meant that their experiences and PTSD symptoms could influence their personality traits and PTSD symptoms. A longitudinal study for people who live in an area where seismic activities are higher may be more worthwhile. However, this means that the survey will risk higher costs and more uncertainty. Moreover, it is vital to check the interrater reliability between rater groups in epidemiological studies, especially those that use other-report questionnaires. However, in our research, the three scales that we used in the study were self-report questionnaires. Before the interviews, we provided written instructions to the interviewers, and their responses clarified the meaning of the question stem. Therefore, the interrater reliability between rater groups was not checked.

## Conclusions

In summary, it has been shown from this study that experiences, personality traits, and genotype influenced the maintenance of PTSD in child survivors who were considered to be followed up without medicine. Postnatal factors are more impactful, whereas LTP genes, such as TrkB, G72, and CNTF, also play essential roles. With the inclination of limited medical resources, targeted measures for specific child survivors could lead to more benefits. In clinical practice, older children with a high tendency toward neuroticism and objective trauma experience a longer duration of symptoms, and they should receive more attention and medical assistance. Individualized psychotherapy aimed directly at personality traits may increase the rate of recovery. The previous section has shown the possible reasons for TrkB, G72, and CNTF in the maintenance of PTSD. Despite providing an equivocal explanation for the relationship and possible reasons, this result anticipates stimulating inspiration for the exploration of directions and research emphases.

## Data Availability

The datasets used and analyzed during the current study are available from the corresponding author on reasonable request.

## Abbreviations

PTSD: Posttraumatic stress disorder
LTP: Long-term potentiation
NMDARs: NMDA receptors
JEPQ: Junior Eysenck Personality Questionnaire
P: Psychoticism
E: Extraversion
N: Neuroticism
